# Beyond Chemical Preservatives: Enhancing the Shelf-Life and Sensory Quality of Ready-to-Eat (RTE) Hummus with Vinegar and Other Natural Antimicrobials

**DOI:** 10.3390/foods12152947

**Published:** 2023-08-04

**Authors:** Layal Karam, Fatma Ghonim, Patricia Dahdah, Grace Attieh, Shama Al-Ahmad, Salma Ghonim, Tareq Osaili

**Affiliations:** 1 Human Nutrition Department, College of Health Sciences, QU Health, Qatar University, Doha P.O. Box 2713, Qatar; 2 Department of Food Science and Technology, Faculty of Arts and Sciences, University of Balamand, Al Koura, Tripoli P.O. Box 100, Lebanon; 3 Department of Agriculture, Section of Agri-Food Biotechnology, University of Sassari, Viale Italia 39/A, 07100 Sassari, Italy; 4 Department of Clinical Nutrition and Dietetics, College of Health Sciences, University of Sharjah, Sharjah P.O. Box 27272, United Arab Emirates; 5 Department of Nutrition and Food Technology, Faculty of Agriculture, Jordan University of Science and Technology, P.O. Box 3030, Irbid 22110, Jordan

**Keywords:** ready-to-eat food, natural antimicrobials, storage, sensory evaluation, spoilage

## Abstract

Hummus is a traditional and very popular Mediterranean ready-to-eat (RTE) food, with growing popularity worldwide. However, it has a high water activity and is susceptible to microbial growth and post-process contamination that limit its quality and shelf-life. For this purpose, the present study compared the use of several antimicrobials, alone or in combination, for hummus preservation during storage (4 °C), for up to 45 days. The chemical preservative potassium sorbate 0.09% (S) was evaluated, along with three natural antimicrobials: garlic 1.25% (G); vinegar 5% (V); natamycin 0.002% (N); or their combination: garlic 1.25%–vinegar 5% (GV); vinegar 5%–natamycin 0.002% (VN); garlic 1.25%–natamycin 0.002% (GN); and garlic 1.25%–vinegar 5%–natamycin 0.002% (GVN) to increase the shelf-life of hummus. A thymol and carvacrol mixture 0.2% (O) was also assessed to preserve and develop a new oregano-flavored hummus. All treatments that included vinegar used alone or in combination had significantly higher antimicrobial effectiveness than the other treatments. They achieved 2.2–3.2, 1.8–3.1, and 1.4–2.1 log reductions in total aerobic counts (TAC), *Pseudomonas* spp., and lactic acid bacteria (LAB), respectively, as compared to the control samples © at day 21. Therefore, the shelf-life of C, S, N, G, GN, and O was around (ca.) 19 days, compared to an extended one of ca. 25 days for V and VN, and ca. 30 days for GV and GVN. Sensory analysis showed the highest acceptability for C, N, S, V, and VN, followed by GV and GVN, and the lowest was for G, GN, and finally O. The findings provide potential alternatives to chemical preservatives, which could be used for natural hummus preservation and shelf-life extension.

## 1. Introduction

In recent years, there has been a global rise in the popularity of ready-to-eat (RTE) foods. This is mainly due to their convenient use, as they require minimal or no preparation prior to consumption and they are easily purchased at supermarkets. Hummus, a chickpea dip, is one of the most popular ready-to-eat foods in the Middle East, and its popularity is growing in the United States and Europe [1]. The global hummus market increased to USD 780 million in 2019 and is predicted to reach USD 910 million by 2024 [2]. In terms of value, the United States and Middle East have the greatest market shares [2]. Hummus is a tahini-based dip made from boiled chickpeas, with the addition of lemon juice or citric acid, garlic, and salt [3]. The preparation starts with soaking the chickpeas in water overnight, then boiling to get a soft texture, and blending with tahina and other ingredients to have a soft mix [4]. Due to its popularity, hummus is being improved industrially to enhance its quality to be healthier and tastier [5]. Hummus is very nutritious, but has a high water activity that supports the growth of several microorganisms, including spoilage microorganisms like *Listeria monocytogenes*, *Salmonella*, and *Escherichia coli* [6]. In addition, it is susceptible to microbial cross-contamination during preparation and post-processing [3]. Those factors limit its production and shelf-life to one or very few days under refrigeration [3]. Some research was conducted on hummus to assess its microbial quality and safety. The microbial safety of RTE hummus was evaluated, with salt being the only preservative [7]. All these studies found that RTE hummus available in supermarkets contained different strains of *Salmonella* and *E. coli.* In addition, the WHO (2008) and Yamani and Al Dababseh (1994) conducted a retail assessment on hummus, and reported that summer hummus had a higher microbial load than winter hummus [3,8]. Olaimat et al. (2017) conducted a study on the growth and inhibition of *Shigella* spp. by using different concentrations of garlic extract and citric acid in hummus stored at different temperatures (4 °C, 10 °C, and 24 °C) [9]. It was reported that *Shigella* spp. were able to survive at the three studied temperatures. In addition, the antimicrobial efficiency was related to the bacterial species, since *Shigella sonnei* was significantly reduced following the use of 2% citric acid or 3% garlic extract compared to *Shigella flexneri*, of which the response was more static. Other studies assessed the microbial quality of hummus when preserved with chemical preservatives and stored at different temperatures [10]. The shelf-life of hummus decreased dramatically when stored at higher temperatures (10 and 15 °C) compared to storing at refrigeration temperature (5 °C), and the combination of potassium sorbate (E202) with citric acid (E330) extended the shelf-life of RTE hummus to 45 days [10].

When prepared on an industrial scale, a new stabilizing and preservation system is required to ensure the safety and quality of RTE products. Canning using commercial sterilization is another common traditional method for hummus preservation, but is undesirable by the consumers and considered highly processed [1,11]. The need for natural hummus preservation has increased to replace chemical preservatives and produce less processed products. Due to increasing consumer awareness concerning chemical preservatives, research is being conducted to replace chemical preservatives with natural food additives having a broad spectrum of antimicrobial effects [12]. Previous studies showed the potential of natural antimicrobials such as garlic powder [9], vinegar [13,14], natamycin [15,16], and oregano essential oils [17,18] in food preservation. However, limited research was carried out to assess the impact of those compounds on microbial spoilage and sensorial acceptance of RTE hummus.

Thus, the purpose of this study was to: (i) compare the antimicrobial effectiveness of potassium sorbate, a commonly used synthetic preservative, with one of natural preservatives like vinegar, garlic, natamycin, thymol, and carvacrol active essential oils, and/or their combinations, and (ii) assess their impact on the microbial shelf-life and sensorial properties of RTE hummus stored at 4 °C for up to 45 days.

## 2. Methodology

### 2.1. Preparation of Hummus Dip

Commercial ready-to-eat hummus was prepared under hygienic conditions using sanitized equipment in an industrial setting, applying good manufacturing practices to simulate real processing conditions of RTE foods. Dry chickpeas were supplied from a local market and soaked in warm water overnight, then boiled with sodium bicarbonate (E500, HAC-01-FS-1, Second House Products, Beirut, Lebanon) and mashed to obtain a smooth chickpea paste. The standard hummus formulation included the following ingredients: cooked and crushed chickpeas (74.46% *w*/*w*), sesame paste (14.36% *w*/*w*), potable bottled water (7.98% *w*/*w*), salt (1.20% *w*/*w*), pure sunflower oil (0.80% *w*/*w*), citric acid (0.80% *w*/*w*), white sugar (0.27% *w*/*w*), and Tara gum (0.13% *w*/*w*). All the formulation ingredients were purchased from the local market, except citric acid (E330, HAC-01-FS-1, Second House Products, Beirut, Lebanon), and they were blended to form the hummus dip. This mixture was used as a control sample.

### 2.2. Preparation of Treatments, Packaging, and Storage of Samples

The following 10 treatments were prepared: control (C) hummus dip with no added antimicrobials; one treatment with potassium sorbate (S), a commonly used synthetic preservative for hummus preservation; three treatments with natural individual antimicrobials, including garlic (G), vinegar (V), natamycin (N); four treatments combining those three natural antimicrobials, including garlic–vinegar (GV), vinegar–natamycin (VN), garlic–natamycin (GN), garlic–vinegar–natamycin (GVN); and one treatment including thymol–carvacrol active essential oil mixture (O). The last mixture was used to develop new oregano-flavored hummus, while achieving antimicrobial preservation. The concentrations of the antimicrobials used are shown below and in Table 1.

- Potassium sorbate (≥99.0%; CAS 24634-61-5; Sigma-Aldrich, Taufkirchen, Germany) was added at a concentration of 0.09% *w*/*w,* which is a standard hummus formulation used in commercial production. This concentration is within the highest acceptable limit for the use of potassium sorbate (0.1%) in preservation of several types of food, as per CODEX ALIMENTARIUS, US, and Canada Regulations [19,20,21].
- Thymol (≥99%, CAS 89-93-8, Sigma Aldrich, Taufkirchen, Germany) and carvacrol (≥98%, CAS 499-75-2, Sigma Aldrich, Taufkirchen, Germany) were mixed in equal amounts (1/1) and added at a concentration of 0.2% (*w*/*w*) [22].
- Natacid^®^ is the commercial name of natamycin (CSK food enrichment, E235, Toruń, Poland). The minimum inhibitory concentration (MIC) of natamycin ranges for fungi between 5 and 20 ppm [23]. The highest range limit (20 ppm) was used in the study (equivalent to 0.002% *w*/*w*) and it is the maximum acceptable limit according to the US and EU regulations [24,25].
- Garlic powder supplied from the local market was added based on its MICs. The MIC of garlic powder ranges from 6.25 to 12.5 mg/mL for 29 enteric bacteria *Bacillus* spp., *Escherichia coli*, *Shigella* spp., *Vibrio* spp., *Salmonella*, *Lactobacillus* spp., etc. [26]. Lower MIC values for pathogenic bacteria and yeasts and molds were obtained by other researchers [27,28]. Based on these data, the highest MIC of 12.5 mg/mL (1.25% *w*/*w*) was used.
- Grapes red vinegar supplied from the local market contains 5% *w*/*w* acetic acid. The MIC of acetic acid against different strains of fungi was higher than that of bacteria and it was found to be 38.1–41.6 mM [29]. The higher end of the range of the antifungal effect of 41.6 mM was used, which is equivalent to 5% *w*/*w* of red vinegar by weight.

Each hummus treatment was poured into plastic pots (Polypropylene, GPI, Beirut, Lebanon), and covered with both a plastic seal (Polyethylene, Flexopress, Beirut, Lebanon) and a plastic cover (Polyethylene, GPI, Lebanon), and then placed into carton boxes (UNIPAK, Beirut, Lebanon) to mimic usual storage conditions. Samples were stored at 4 °C throughout the experiment and analyzed until the end of their shelf-life or over a period of 45 days.

### 2.3. Microbiological Analysis

Samples (25 g) of hummus were aseptically transferred into individual stomacher bags (Interscience, Saint-Nom-la-Bretèche, France) and homogenized with 225 mL of sterile buffered peptone water (0.1%) in a stomacher (Lab Blender 400, Seward Medical, West Sussex, United Kingdom ) for 120 s, in order to prepare the serial ten-fold dilutions.

The enumeration of the total aerobic count was performed on a plate-count agar (HiMedia, Kennett Square, United States) and incubated at 37 °C for 48 h [30,31]. The enumeration of *Pseudomonas* spp. was performed on a *Pseudomonas* agar base supplemented with CFC (HiMedia, Kennett Square, United States) and incubated at 37 °C for 48 h [32,33]. The enumeration of yeasts and molds was performed on a Sabouraud dextrose agar (HiMedia, Kennett Square, United States) and incubated at 25 °C for 5 days [34]. The enumeration of *S. aureus* was performed on a Baird Parker agar (HiMedia, Kennett Square, United States) and incubated at 37 °C for 48 h [35,36]. The enumeration of lactic acid bacteria was performed on an MRS agar (HiMedia, Kennett Square, United States) using the pour plate overlay technique, and the plates were incubated at 30 °C for 72 h [37].

The qualitative analysis of *Salmonella* was performed on a *Salmonella Shigella* agar (SS Agar, Modified, M1032, HiMedia, Kennett Square, United States) and incubated at 37 °C for 24 h after pre-enrichment with BPW (0.1%) at 37 °C for 20 h, and an enrichment using the Rappaport Vasiliadis broth (Rappaport Vassiliadis *Salmonella* Enrichment Broth, MH1491, HiMedia, Kennett Square, United States) for 24 h at 42 °C. Presumptive *Salmonella* colonies were identified using a serological test consisting of poly-H antigen agglutination with Poly-O (Remel, Thermo Fisher, MA, USA) and a biochemical test consisting of an API 20E test kit (BioMerieux, Craponne, France) [38]. Both *S. aureus* and *Salmonella* were absent from all samples during the test period. For the remaining microorganisms, plates having 25–250 colonies were counted, and microbiological data were converted into logarithms of the number of colony-forming units (CFU/g) ± standard deviation (SD).

### 2.4. Sensory Analysis

A consumer acceptance test was conducted as suggested by Wangcharoen and Phimphilai (2016) [39]. About 20 g of each hummus sample was served at refrigeration temperature (4 °C) with a random three-digit code number. For every test point, 10 untrained panelists, aged between 20 and 64 years, were requested to taste the hummus samples. Throughout the test period, neither *S. aureus* and *Salmonella* were detected in any of the samples. As a result, the hummus samples were suitable for sensorial analysis. The scores were recorded according to a 9-point hedonic scale for appearance, odor, taste, and overall liking, with 1 being the least liked and 9 the most liked. Sample having a score <6 for any of the attributes were considered unacceptable. The panelists were requested to drink water between the samples. 

### 2.5. Statistical Analysis

Experiments were conducted in duplicate (n = 2) and tests in three replicates. The results were reported as the mean values +/− standard deviation (SD). Microbiological counts and sensory scores were analyzed on SPSS version 25 (IBM Corp. Released 2017. IBM SPSS Statistics for Windows, Version 25.0. Armonk, NY, USA: IBM Corp). A one-way ANOVA was used to analyze the variance. The difference among the means was tested via LSD (least significant difference), and statistical significance was considered at *p* < 0.05.

## 3. Results and Discussion

### 3.1. Microbiological Analysis

#### 3.1.1. Total Aerobic Count (TAC)

**Figure 1:**
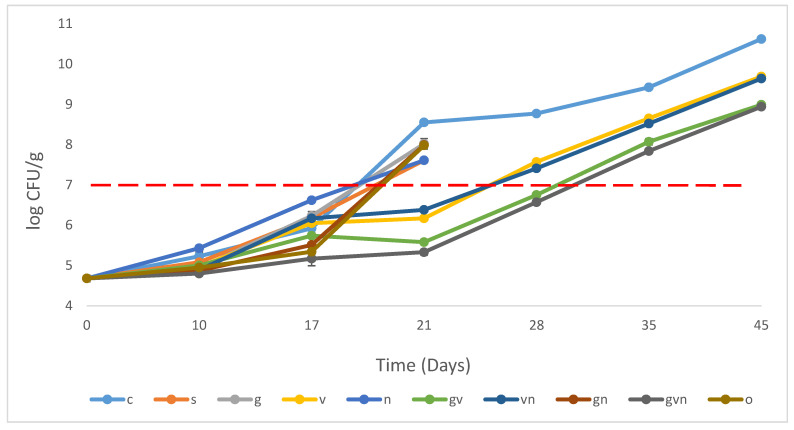
Population increase in TAC in hummus samples stored at 4 °C for 45 days for the following treatments: control (C), potassium sorbate (S), garlic (G), vinegar (V), natamycin (N), garlic and vinegar (GV), vinegar and natamycin (GN), garlic and natamycin (GN), garlic, vinegar, and natamycin (GVN), thymol–carvacrol mixture (O). The horizontal line (-----) corresponds to the cut-off microbiological value (end of shelf-life), and the error bars indicate the standard deviations.

The total aerobic count is a general indicator of the quality and shelf-life of hummus. Figure 1 illustrates the growth pattern of TAC in all hummus treatments over different days among different treatments. At day 0, the initial value of all hummus samples was 4.7 CFU/g, highlighting a good quality based on the maximum acceptable limit of 7.0 log CFU/g for TAC in hummus dipping [40,41,42]. At day 21, all the samples, i.e., C, S, N, G, GN, and O, reached the maximum acceptable limits for TAC of 7 log CFU/g [40,41,42], except vinegar treatments (V, VN, GV, GVN). The antimicrobial effect of vinegar in hummus was proven superior to other treatments as TAC values were significantly lower in the samples treated with vinegar than the control and other treatment samples (*p*-value < 0.001). Those four vinegar treatments showed 2.2–3.2 log reduction compared to the control samples at day 21. No significant difference (*p*-value > 0.05) was observed between potassium sorbate (S) and natamycin (N) treatments that had a modest reduction of 0.9 log compared to the control. In addition, garlic (G and GN) and active essential oil component (O) treatments had similar slight antimicrobial activity (*p*-value > 0.05), with a log reduction of 0.5–0.6 compared to the control. The shelf-life of all those treatments (C, S, N, G, GN, and O) ended at ca. 19 days. For this reason, only the samples treated with vinegar were studied for 45 days to determine their shelf-life in comparison to the control (C). The maximum acceptable limit was exceeded on day 28, when vinegar (V) was used alone or in combination with natamycin (VN). However, when garlic was added (GV and GVN), this limit was exceeded on day 35. A shelf-life of ca. 25 days was thus achieved in treatments (V) and (VN), while (GV) and (GVN) resulted in an extended shelf-life of ca. 30 days. On day 45, GV and GVN had significantly higher log reductions of TAC (*p*-value < 0.05) than V and VN, of 1.6–1.7 and 0.9–1, respectively, compared to the control samples. A synergistic effect was observed when garlic was combined with vinegar, leading to the highest effectiveness in reducing TAC for GV and GVN. However, a lower antibacterial effect was observed when natamycin was combined with either vinegar (V and VN) or garlic (G and GN) treatments, most probably due to the antifungal properties of natamycin [16]. Overall, the lowest TAC count or highest log reduction was mainly recorded for GVN compared to all the other treatments during the test days. Several studies showed that combining antimicrobials has more effect against different types of microorganisms in hummus and ready-to-eat food due to the wider spectrum of activity and synergistic activity of antimicrobials such as the combination of potassium sorbate and sodium metabisulfite [10], natamycin, and chitosan-based films [15], natamycin and chitosan [43], garlic and chitosan [44], citric acid and garlic [9]. When the same thymol and carvacrol mixture was used at a higher concentration of 0.8%, a higher effectiveness was achieved in controlling TAC counts in marinated beef (1.1–1.3 logs reduction) [17] and marinated chicken (2.9–3 logs reduction) [18]. Another study showed that using 2% active essential oil components thymol, carvacrol, and cinnamaldehyde in combination with yogurt-based marinade at 4 and 10 °C had significant log reductions, of 3.6–4.4 log CFU/g and 1.0–2.7 log CFU/g, respectively, against *E. coli* O157:H7 and *Salmonella* compared to untreated samples of camel meat chunk [45]. These active components showed similar significant antimicrobial effects against *E. coli* O157:H7 and *Salmonella* when added to chicken tawook at 1% [45]. This highlights the importance of both the concentration of antimicrobials used and the effect of the type of food matrix on antimicrobial efficiency.

Regarding the other individual antimicrobials used, the limited antibacterial effects of potassium sorbate and natamycin used alone can be related to their higher effectiveness against yeast and molds compared to bacteria [46]. Another study showed that the addition of natamycin to Greek salad incubated at 4 °C had an insignificant effect on reducing TAC (*p*-value > 0.05) [16]. Potassium sorbate was previously added at 0.05% and 0.1% to hummus samples, and extended the shelf-life of hummus to 35 and 49 days, respectively, at 5 °C, compared to the control [10]. The variation between the results of the current study and the previous one can be due to several factors such as potassium sorbate combination with 10% citric acid in hummus samples to increase the efficiency of the treatments, the difference in concentrations of used antimicrobials, and the difference in hummus formulation.

With respect to the use of garlic powder, the present results are in agreement with those of previous studies showing that using garlic powder had no effect on the microbial load when added to rabbit meat burgers [47]. Other studies showed that using garlic powder on camel meat has kept the TAC below the maximum permissible limit (MPL) of 7 log CFU/g during the first 7 days, and lowered the TAC significantly until day 14 compared to the control. However, the treated sample with garlic exceeded the MPL of 7 log CFU/g at day 14, which indicates a weak antimicrobial effect of garlic powder [48]. Similar to our findings, Olaniran et al. (2015) reported that garlic powder had greater effectiveness when used with other antimicrobials in inhibiting microbial growth in tomato paste [49].

The observed inhibitory effect of vinegar against TAC in RTE hummus was similarly proven by other studies [46,50]. In a carp fillet treated with a concentration of 3.13 mg/mL buffered vinegar for 9 days and stored in ice, a reduction of more than 1 log CFU/g for TAC was achieved compared to the control [14]. In addition, TAC did not exceed 4 CFU/g at day 6 or 6 CFU/g at day 9, which indicates the effectiveness of vinegar to preserve the food [14]. Another study by Karapinar and Gönül (1992) showed that soaking parsley in 1, 2, and 5% acetic acid solutions for at least 15 min decreased mesophilic aerobic bacteria by 5 logs CFU/g [13].

#### 3.1.2. *Pseudomonas* spp.

**Figure 2:**
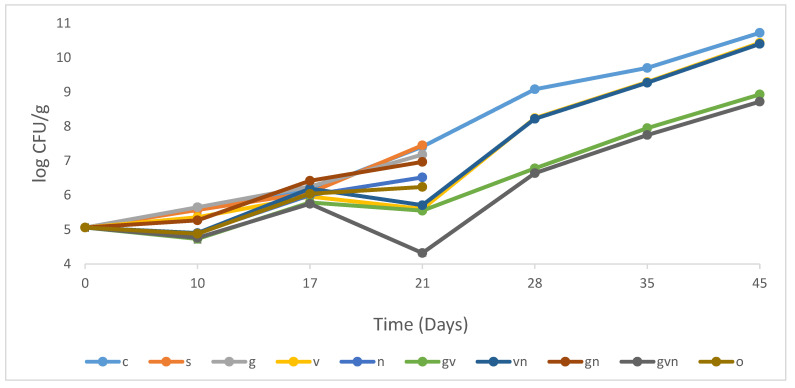
Population increase in *Pseudomonas* spp. in hummus samples stored at 4 °C for 45 days for the following treatments: control (C), potassium sorbate (S), garlic (G), vinegar (V), natamycin (N), garlic and vinegar (GV), vinegar and natamycin (GN), garlic and natamycin (GN), garlic, vinegar, and natamycin (GVN), thymol–carvacrol mixture (O). The error bars indicate the standard deviations.

*Pseudomonas* spp. are Gram-negative, psychrotrophic bacteria that are able to grow between 3 and 7 °C [51]. The inhibitory effect analysis of different antimicrobial treatments on *Pseudomonas* spp. In hummus are given in Figure 2. Potassium sorbate treatment had no antimicrobial effect, and no significant difference (*p* > 0.05) was observed between this treatment and the control during all the tested days. The individual antimicrobial (S, N, G, GN, and O) treatments were assessed until 21 days (final test day at the end of shelf-life). At day 21, a slight antimicrobial effect was noted for natamycin and garlic treatments (G and GN) that showed 0.2–0.9 log reductions compared to the control. A higher significant effect (*p* < 0.05) was noted for thymol and carvacrol, that achieved a 1.2 log reduction. Similar to TAC, the highest significant antimicrobial effect (*p* < 0.05) was observed for treatments including vinegar (V, VN, GV, GVN) that had 1.8–3.1 log reductions as compared to the control (day 21). At day 45, the log reductions were 0.3 for V and VN, 1.8 for GV, and 2 for GVN treatments. The lowest *pseudomonas* count and the highest efficiency was mostly for GVN treatment during all the test days. Those results highlight the synergetic effect of garlic with vinegar and the importance of antimicrobial combination for the control of *Pseudomonas*. Similarly, a study performed by Stanojevic et al. (2009) showed that preservatives (sodium nitrite + potassium sorbate) work more effectively against microorganisms when used in combination than alone [52]. Furthermore, the combination of rosemary extract with buffered vinegar had antibacterial activity against *Pseudomonas fragi* and *P. psychrophilia* compared to the control [14]. Chouliara et al. (2007) reported that the most effective way to reduce *Pseudomonas* spp. counts is a combination of antimicrobial treatments (oregano essential oil) and a reduction in O_2_ levels achieved via modified atmosphere packaging [53]. Different studies focusing on plant-extracted antimicrobials discussed the effectiveness of using oregano oil and thyme oil to control the growth of different species of Gram-positive and Gram-negative bacteria, as well as fungi [50,54,55] Lobiuc et al. [50]. discussed the role of phenolic compounds in the alteration of bacterial structure and metabolism.

In other studies, the effectiveness of sorbate against *Pseudomonas* spp. was dependent on several factors such as the pH of the medium, the concentration used, the specific *Pseudomonas* species, and the varying minimum inhibitory concentrations associated with different species [52,56,57]. Previous studies showed that garlic was helpful in combating a wide range of Gram-positive and Gram-negative bacteria, including *Pseudomonas* spp. [58]. Other research confirmed that applying garlic powder to camel meat significantly reduced *Pseudomonas* until day 14 compared to the control, keeping *Pseudomonas* below the maximum permissible limit (MPL) of 7 log CFU/g for the first 7 days. However, the garlic-treated sample exceeded the MPL of 7 log CFU/g at day 14, indicating that garlic powder’s antibacterial action was poor [48]. Another study showed that using garlic alone or in combination with other essential oils such as thyme, rosemary, and cinnamon had a strong antimicrobial activity against foodborne pathogens in meat and meat products [59]. Concerning, natamycin, no effect was shown on inhibiting the growth of Gram-negative bacteria including *Pseudomonas* spp. due to its antifungal properties [60,61]. However, natamycin contributed to an extended shelf-life of tzatziki, a ready-to-eat traditional Greek deli salad, and thus a lower *Pseudomonas* count, when combined with citrus extracts and vacuum packaging, due to a synergistic effect between the three treatments [16].

In a study on ready-to-eat meat products, films containing oregano extracts were used, and *Pseudomonas* spp. were similarly reduced by 0.95 logs when compared to the control sample [62]. The efficiency of vinegar was also demonstrated in in vitro tests, where cider vinegar, red vinegar, and white vinegar all had an inhibitory impact on the bacterium that causes tomato bacterial speck (*Pseudomonas syringae* pv. tomato) [63].

Spoilage is detected in RTE foods when *Pseudomonas* spp. reach 7–8 Log (CFU/g) [42]. The initial *Pseudomonas* counts were 5.5 log CFU/g and none of the 10 treatments reached 8 logs at day 21. Samples C, V, and VN reached this limit at day 28 and the remaining GV and GVN treatments exceeded this value at day 45. *Pseudomonas* spp. is a major reason behind the spoilage of chilled RTE foods [64]. Spoilage is caused by enzymes produced by *Pseudomonas* spp., such as lipases and lyases, during cold storage [65].

#### 3.1.3. Lactic Acid Bacteria (LAB)

**Figure 3:**
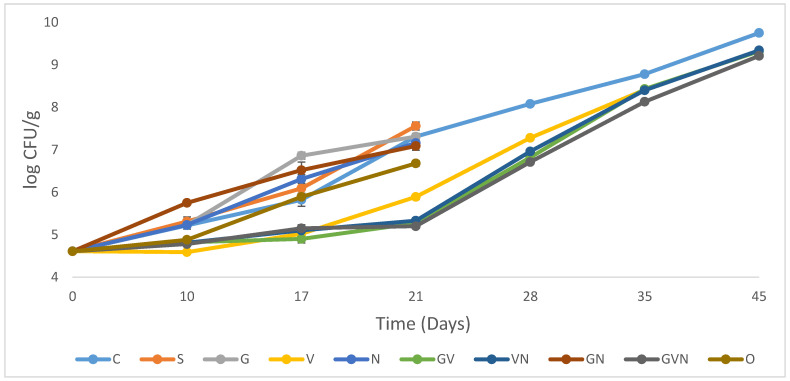
Population increase in lactic acid bacteria in hummus samples stored at 4 °C for 45 days for the following treatments: control (C), potassium sorbate (S), garlic (G), vinegar (V), natamycin (N), garlic and vinegar (GV), vinegar and natamycin (GN), garlic and natamycin (GN), garlic, vinegar, and natamycin (GVN), thymol–carvacrol mixture (O). The error bars indicate the standard deviations.

Lactic acid bacteria (LAB) are found abundantly in several fermented foods, but they cause spoilage in non-fermented products like several types of ready-to-eat products and hummus [66,67]. The primary cause of deterioration driven by LAB is the creation of metabolites that result in undesirable modifications to the substrate appearance, texture, and flavor [68]. Figure 3 shows the growth of LAB among different treatments during the storage period. LAB in hummus treatments followed a similar trend, with an increase in counts throughout the days of storage. Garlic (G) and potassium sorbate (S) treatments showed no antimicrobial effect against LAB. Conversely, S treatment showed a significantly higher LAB count (*p* < 0.05) than the control at day 21. Potassium sorbate might have provided a suitable environment for LAB growth by inhibiting other competitors like other types of bacteria, yeasts, and molds. When natamycin was used alone (N) or in combination with garlic (GN), a minimal log reduction of 0.1–0.2 was noted at day 21, as compared to the control. Thymol and carvacrol had a significantly higher (*p* < 0.05), but a modest, reduction of LAB counts, by only 0.6 logs. Similarly, vinegar treatments were the most effective treatments (*p* < 0.05) against LAB, achieving 1.4 to 2.1 log reduction (day 21), with GVN recording the lowest LAB counts. The absence of garlic antimicrobial activity, when used alone, was also reflected by a lower synergetic effect when combined with vinegar or natamycin against LAB. The antimicrobial combination related to the four vinegar treatments thus had a lower effectiveness of 0.4–0.5 log reductions (compared to control) at day 45 (as compared to the higher observed effect on TAC and *Pseudomonas*). In addition, the lower antimicrobial activity generally observed for all the treatments can be also related to the low pH of Hummus formulations (4.5–4.8) and the higher tolerance of LAB to low pH conditions, as compared to TAC and *Pseudomonas*. Previous studies demonstrated that LAB isolated from fermented food sources like *Pediococcus acidilactici* and *Pediococcus pentosaceus* were resistant to acidic conditions [69]. Similarly, when garlic extract was added to ferment green olives, lactic acid bacteria were not suppressed, and only a minor inhibition was seen at a high concentration of 5% [70]. As for potassium sorbate, its addition to seasoned olives did not appear to impact LAB, and only high concentrations of 1000 to 2000 ppm can cause a minor inhibition of lactic acid bacteria [71]. In addition, only when the potassium sorbate level was raised to 15% in sweet potato films, a significant suppression of *E. coli* was detected [72]. As for vinegar, previous research in chicken retail cuts has indicated that the addition of 1.0% vinegar treatment similarly decreased the LAB counts compared to the control treatments at days 12 and 16 [73]. Concerning the use of active essential oils components, Chouliara et al. (2007) reported that the most effective treatment against LAB was a concentration of 1% of oregano essential oils combined with modified atmosphere packaging (MAP) [53]. Similarly, a study by Skandamis and Nychas (2001) indicated that under all packaging conditions (air, modified atmosphere), oregano oil, with concentrations of 0.5% and 1%, was effective in controlling LAB in minced meat [74]. Such high concentrations were not used in this study due to limited sensorial acceptability of the product. Furthermore, the addition of 0.5%–1.5% thyme essential oil to hummus decreased *Salmonella* by 1.0–2.9 log CFU/g at 4 °C over the course of 10 days [75]. It was also shown that using 0.17% and 0.35% thyme oil had a great effect in controlling *E. coli* when added to soft cheese [76]. Another study showed that the combination of antimicrobials like 1% garlic and 0.5% chitosan in hummus was able to reduce *Salmonella*, *E. coli* O157:H7, and *L. monocytogenes* by 0.7–2.5, 0.6–2.2, and 1.0–1.5 log CFU/g, respectively [44]. This shows the interest of using essential oils, garlic, and similar antimicrobial combinations for the control of both spoilage and safety, especially for pathogens such as *Salmomella* and *Listeria Monocytogenes* known to be significant hazards in hummus [77].

Lactic acid bacteria are Gram-positive bacteria that that use carbohydrate fermentation to generate lactic acid. LAB are psychrotrophic bacteria that have adapted to industrial environments, and can thus contaminate foods easily [78]. Spoilage related to lactic acid production can start when LAB levels reach 6 log, and will eventually be clearly detected at high levels of 9 log [42,79]. The initial LAB counts were 4.6 log (CFU/g) at day 0 and the high levels of 9 logs were not reached in any of the 10 treatments at day 21, but were exceeded in all the remaining 5 treatments at day 45.

Yeasts and molds were only detected in the control samples after day 10 at low levels of around 4 log CFU/g, showing the efficiency of the antimicrobials used in controlling fungal growth [44] and the poor competition of yeasts and molds with bacteria like TAC, *Pseudomonas* spp., and LAB that dominated the microbiota of the hummus samples [17].

In summary, all the individual antimicrobial treatments (S, N, G), except vinegar (V), the thymol–carvacrol mixture, and antimicrobial combination using only garlic and natamycin (GN), showed poor or limited antimicrobial efficiency in hummus preservation at the concentrations used. However, all the treatments using vinegar alone (V) or in combination with other antimicrobials (VN, GV, GVN) had superior significant antimicrobial activity compared to the control and other treatments, with the highest efficiency recorded for the GVN combination. The pH values of the formulations (C, S, G, N, GN, and O) ranged between 4.7 and 4.8. However, hummus samples treated with vinegar had lower pH values that ranged between 4.5 and 4.6. The remarkable antimicrobial activity of vinegar is related to the presence of acetic acid. This weak organic acid has greater ability to penetrate the cells when it is in a non-charged state (undissociated fraction) compared to its dissociation products [80]. The undissociated form of acetic acid enters the bacterial cell membrane, causing the accumulation of the proton H+ which causes the increase in acidity of the cell cytoplasm. This causes cell damage, enzyme denaturation, DNA/RNA synthesis alteration, and energy depletion for the bacterial cells, leading to alteration in cell growth and the cause of cell death [81]. Weak acids also contribute to an increase in the lag phase [29]. In addition, the slight decrease in pH from 4.7–4.8 (control and other treatments) to 4.6–4.5 after vinegar addition can contribute to an increased proportion of the undissociated fraction of acid and the increased antimicrobial activity observed for samples containing vinegar [82]. Natamycin is a microbial derived from *Streptomyces natalensis* and *Streptomyces chattanoogensis* bacteria, and is usually used as an antifungal ingredient by binding with the fungal cell membrane and altering its permeability [83]. It is on the FDA Generally Recognized as Safe (GRAS) list and the European Food Safety Authority (EFSA) concluded that it is poorly absorbed in the gastrointestinal tract, which allows for an adequate margin of safety in current use [24,84]. Furthermore, low concentrations of natamycin were proven to inhibit mycotoxin production by toxigenic molds [85]. Garlic is also on the GRAS list approved by the FDA to be used as an antimicrobial [86]. It is reported that garlic contains several active compounds such as allicin, that showed antimicrobial effect against Gram-positive and Gram-negative bacteria [87]. Garlic can alter bacterial proteins and inactivate bacterial enzymes, leading to bacterial death [88]. Thus, the combination of natural bactericides such as garlic and vinegar, and natural fungicide agent like natamycin, is a great hurdle approach to increase the synergy among antimicrobials and control both bacteria and fungi in food applications.

### 3.2. Sensorial Analysis

The sensory characteristics (appearance, odor, taste, overall liking) of hummus treated with different antimicrobials are summarized in Table 2. The results are presented at day 0 after formulation and at day 21, which is the last test point where all the treatments were tested and compared since after this day, six treatments had exceeded their microbial shelf-life.

For appearance, all the treatments were acceptable at both days 0 and 21. At day 0, no significant difference (*p*-value > 0.05) was noted among the treatments, and at day 21, a significant difference (*p*-value < 0.05) was noted between natamycin and sorbate, showing the highest scores for appearance compared to the lowest ones for garlic (G) and garlic–natamycin (GN). For odor, all treatments were sensorially acceptable at day 0, but all garlic treatments (G, GV, GN, GVN) and O had lower odor scores (6.1–6.6) compared to higher ones for C, S, N, V, and VN (7.3–7.8). At day 21, all the treatments remained acceptable for odor except G, GN, and O that were below the acceptable scores (˂6). For taste, all the treatments were acceptable at day 0, except the thymol–carvacrol (O) formulation that had a score below 6 and was significantly lower than all the other formulations (*p*-value < 0.05). At day 21, similar to the odor trend, G, GN, and O were unacceptable and had significantly lower scores than the other treatments (*p*-value < 0.05). For the overall liking, similar to the taste trend, only O was unacceptable at day 0, while both O and G were not liked at day 21 (score < 6). In general, and taking into account all the sensory attributes, the treatments (C, S, N, V), and VN) had mostly the highest scores. A lower score was generally noted for treatments GV and GVN. However, the former seven treatments were sensorially acceptable for all the evaluated parameters at both days 0 and 21. The least liked treatments were G, that was unacceptable for odor, taste, and overall at day 21, and GN, that was unacceptable at day 21 for both odor and taste. Finally, O had the lowest scores among all the treatments and was unacceptable for odor only at day 21, and for the taste and overall attributes at both days 0 and 21. It can be concluded that garlic addition reduced the sensorial acceptability of the hummus samples probably due its strong and sharp flavor and odor, but its combination with other antimicrobials, mainly vinegar or both vinegar and natamycin, helped reduce or mask the effect of garlic and improved its acceptability. This result agrees with another study that showed that when minced fresh garlic was added to hummus, a lower level of acceptance regarding the flavor was noted compared to the control [44]. Moreover, Khoshtinat et al. (2022) concluded that garlic cannot be used directly in salad dressing production due to its intense odor [89]. However, adding lemon juices in high concentrations to the chickpea gel hid the smell of garlic and other ingredients [90]. In addition, G and GN reduced the appearance score of hummus and had a darker yellow color compared to the other treatments; this can be related to the yellow color of garlic powder. Similarly, a previous study showed that the addition of garlic powder had an increase in yellowness of rabbit burgers [47]. However, adding minced garlic to chickpea gel had an insignificant influence on color, but the general appearance was affected due to increasing consistency, showing white spots with air pockets compared to the control samples that appeared smoother and creamier [90]. Interestingly, formulation using vinegar improved the scores and reduced the negative effect of garlic probably due to the presence of acetic acid resulting in lighter appealing color for hummus samples. A previous study showed that using low-pH acid marination for beef steak caused denaturation of muscle proteins, resulting in the lighter color for beef tissues [91]. In addition, the acidic flavor provided by vinegar was a desirable combination with the hummus dip formulation, showing that food acids can be added to give a sharper flavor to food in addition to their antimicrobial and antioxidant effect [92]. A previous study on a Portuguese traditional ready-to-eat meat treated with vinegar showed that sensory acceptability was not diminished by vinegar. Despite the lack of any discernible differences, vinegar samples typically received better ratings for overall appreciation [93]. The lowest sensorial scores observed for the thymol–carvacrol combination showed that despite the relatively low concentration used, consumers reported poor acceptability for the new oregano-flavored hummus, and they preferred the traditional original taste. Comparably, Mexis et al. (2009) showed that when oregano essential oil was applied to cod fish roe at minimum concentrations of 0.1% (*v*/*w*), a strong flavor was recorded [94]. Thus, formulations using essentials oils for preservation are always limited by sensorial preferences. On another note, the similarity between sensorial scores and microbial efficiency of potassium sorbate and natamycin, may suggest that natamycin, as a natural preservative, can replace the synthetic preservative potassium sorbate to provide a natural formulation, but with a limited preservation effect. However, taking into account both microbiological quality and sensorial acceptability, all the treatments containing vinegar (V, VN, GV, GVN) provide a potential interesting option for preservation and shelf-life extension.

## 4. Conclusions

Hummus is of a Mediterranean origin, but is becoming popular globally. Shelf-life extension, high quality, and safety standards are currently critical requirements to meet industrial-scale production. This study showed the high potential of using vinegar and its combination with other preservatives for the preservation of microbial quality, without compromising the sensorial properties of hummus. This will provide potential preservation methods for hummus quality improvement and shelf-life extension. Furthermore, it can be an excellent alternative to replace chemical preservatives and canned processed products that are highly undesirable by the consumers. Garlic, vinegar, and natamycin, as natural antimicrobials, present economically viable and sustainably sourced solutions for food preservation in the industry. The results underlined the importance of natural preservatives in extending the shelf-life of hummus, and future studies are needed to assess other treatments such as novel technologies, physical-based preservation, and other antimicrobial formulations and their impact on the quality and safety of the product.

## Figures and Tables

**Table 1:** Different concentrations of individual antimicrobials and their combination used in the preparation of the hummus samples.

Label	Antimicrobial Treatment	Concentration % (*w*/*w*)
C	No antimicrobials added	N/A *
S	Potassium sorbate	0.09
G	Garlic powder	1.25
V	Vinegar	5
N	Natamycin	0.002
GV	Garlic powder + vinegar	1.25 + 5
VN	Vinegar + natamycin	5 + 0.002
GN	Garlic powder + natamycin	1.25 + 0.002
GVN	Garlic powder + vinegar + natamycin	1.25 +5 + 0.002
O	Mixture of active components of essential oils at equal amounts (thymol + carvacrol)	0.2

* No added antimicrobials.

**Table 2:** Sensory analysis of appearance, odor, taste, and overall attributes for the following treatments: control (C), potassium sorbate (S), garlic (G), vinegar (V), natamycin (N), garlic and vinegar (GV), vinegar and natamycin (GN), garlic and natamycin (GN), garlic, vinegar, and natamycin (GVN), thymol–carvacrol mixture (O).

Sensorial Analysis Score (Mean ± SD)								
Treatment	Appearance		Odor		Taste		Overall	
	Day 0	Day 21	Day 0	Day 21	Day 0	Day 21	Day 0	Day 21
**C**	7.9 ± 0.3 ^a^	8.3 ± 0.7 ^ac^	7.6 ± 0.7 ^a^	7.9 ± 1.8 ^a^	7.6 ± 0.8 ^a^	7.4 ± 1.6 ^a^	7.8 ± 1.0 ^a^	7.7 ± 1.9 ^a^
**S**	8.4 ± 0.8 ^a^	8.7 ± 0.5 ^a^	7.8 ± 1.3 ^a^	7.9 ± 1.1 ^a^	7.9 ± 1.2 ^a^	7.6 ± 1.8 ^ab^	7.9 ± 1.3 ^a^	7.7 ± 1.2 ^a^
**G**	8.1 ± 1.0 ^a^	7.9 ± 1.4 ^bc^	6.1 ± 1.8 ^c^	**5.4 ± 1.5 ^b^**	6.6 ± 1.6 ^ab^	**5.1 ± 1.3 ^c^**	6.0 ± 1.9 ^b^	**5.9 ± 1.1 ^b^**
**V**	8.4 ± 1.1 ^a^	8.4 ± 0.7 ^ac^	7.4 ± 1.3 ^ad^	7.4 ± 1.4 ^ac^	7.6 ± 1.3 ^a^	8.3 ± 0.8 ^ad^	7.6 ± 1.3 ^ac^	8.1 ± 1.0 ^a^
**N**	8.4 ± 0.5 ^a^	8.7 ± 0.5 ^a^	7.6 ± 0.8 ^a^	8.6 ± 0.7 ^ad^	7.9 ± 1.0 ^a^	8.6 ± 0.7 ^bde^	8.0 ± 0.7 ^a^	8.6 ± 0.5 ^a^
**GV**	8.2 ± 1.0 ^a^	8.3 ± 0.9 ^ac^	6.1 ± 1.1 ^c^	6.5 ± 1.4 ^bce^	6.6 ± 1.0 ^ab^	6.5 ± 1.2 ^a^	6.6 ± 1.2 ^bc^	6.7 ± 1.1 ^ab^
**VN**	8.5 ± 0.7 ^a^	8.3 ± 0.7 ^ac^	7.3 ± 0.9 ^ae^	6.1 ± 2.1 ^bf^	7.3 ± 0.7 ^a^	7.8 ± 0.4 ^ae^	7.9 ± 0.7 ^a^	7.0 ± 1.8 ^ab^
**GN**	7.9 ± 1.1 ^a^	7.4 ± 0.8 ^bc^	6.4 ± 1.2 ^cde^	**5.1 ± 0.7 ^b^**	6.9 ± 1.5 ^a^	**4.9 ± 0.6 ^c^**	6.6 ± 1.1 ^bc^	6.6 ± 1.2 ^ab^
**GVN**	7.9 ± 1.0 ^a^	8.1 ± 0.3 ^ac^	6.4 ± 1.3 ^cde^	6.7 ± 1.2 ^adef^	6.8 ± 1.5 ^a^	6.6 ± 0.5 ^a^	6.1 ± 0.7 ^b^	7.3 ± 1.2 ^ac^
**O**	8.6 ± 0.7 ^a^	8.4 ± 0.7 ^ac^	6.6 ± 1.9 ^abc^	**5.2 ± 1.9 ^b^**	**3.8 ± 1.9 ^c^**	**3.1 ± 2.0 ^f^**	**4.1 ± 1.7 ^d^**	**4.7 ± 1.9 ^b^**
***p*-value**	**0.485**	**0.018**	**0.008**	**<0.001**	**<0.001**	**<0.001**	**<0.001**	**<0.001**

mean ± SD; *p*-value < 0.05 is considered significant; Columns with different superscripts differ, *p*-value < 0.05; Values in bold and underlined are below the acceptable sensorial score (score < 6).

## Data Availability

The datasets generated for this study are available on request to the corresponding author.
